# Prehospital stroke care in Africa: The reality and potential solutions

**DOI:** 10.1111/cns.14005

**Published:** 2022-11-01

**Authors:** Rita Melifonwu, Ikenna Onwuekwe, Jing Zhao, RenYu Liu

**Affiliations:** ^1^ Life after Stroke Centre Stroke Action Nigeria Onitsha Nigeria; ^2^ Department of Nursing Science Nnamdi Azikiwe University Awka Nigeria; ^3^ Department of Medicine, Faculty of Medical Sciences, College of Medicine University of Nigeria Nsukka Nigeria; ^4^ Department of Neurology Minhang Hosptial, Fudan University Shanghai China; ^5^ Department of Anaesthesiology and Critical Care Perelman School of Medicine at the University of Philadelphia Philadelphia Pennsylvania USA; ^6^ Department of Neurology Perelman School of Medicine at the University of Pennsylvania Philadelphia Pennsylvania USA

Available reports show that stroke is the second highest cause of mortality globally, resulting in 6.2 million deaths a year. Two‐thirds of all strokes occur in developing countries. Africa appears to have the highest incidence, prevalence, and case fatality rates of stroke, as reported in several studies.^1^ This unfortunate situation is attributed to population growth, unbalanced development, and increasing western diets, resulting in a rise in vascular disease risk factors that are largely modifiable through education. These risk factors including smoking, physical inactivity along with unhealthy diets, thus invariably resulting in increased prevalence of hypertension.^1^ Owolabi et al have documented Africa to have the highest burden of hypertension, which is the most common modifiable risk factor for stroke.^2^

In many countries of Africa, stroke is the most prevalent medical and neurological cause of inpatient hospitalization.^3^ Sub‐Saharan Africa (SSA) bears the highest burden of stroke worldwide with age‐standardized stroke incidence rates of up to 316 per 100,000, prevalence rates of up to 14 per 1000 population, and 1‐month fatality rates of up to 40% with hospitalization of stroke patients ranging from 0.9% to 4.0%. In Nigeria, the current prevalence of stroke is reported as 1.14 per 1000 while the 30 days case fatality rate ranges from 21% to 45%.^4^ Most of the mortalities were within the acute phase. In 2005, the unacceptable level of acute stroke care in the medical facilities across Nigeria and Africa was addressed to reverse or mitigate the devastating effects, which are best handled within the golden period of 3–4.5 h. Nearly 2 decades on, little has changed in this landscape in terms of prehospital stroke care in much of Africa.

Within the framework of prehospital stroke care, the use of mobile stroke units (MSU) has developed, with imaging technology use in the ambulance. This enables quicker access with possible immediate treatment, emergency department bypass, and direct admission to the acute stroke unit. In 2003, mobile stroke units emerged in rural Germany to bring expert stroke care quickly to the patient and since then MSUs have expanded to include urban settings. MSUs prevent in‐hospital treatment delays, and most MSU programs are activated on scene by first responders when stroke symptoms are recognized. However, the suitability to deploy MSU in Africa remains uncertain due to the high cost of maintenance insufficient manpower and lack of proper trainings. The current challenges include the fact that even regular ambulance service is inadequate in many regions in Africa. Training for healthcare personnel and volunteers for stroke care are urgently needed.

The last point is very critical as a target for public health education in Africa. The first step is to increase the ability of the average individual (child or adult) to quickly recognize the key signs and symptoms of stroke and to call for emergency medical attention immediately. The current educational videos and other educational materials developed and dispersed by the non‐governmental organization Stroke Action Nigeria and collaborators, in multiple local languages, is a great step in enhancing prehospital stroke care in rural and urban settings of Africa. Here, we strongly advocate the novel strategy of Stroke 112 developed recently.^5^ The strategy of Stroke 112, using the emergency phone number to represent stroke signs and symptoms and to immediately call 112, is an innovative way to overcome language barriers, since Africa has a large variety of languages. A similar strategy used in China demonstrated huge success in improving stroke prehospital care and reducing prehospital delay.^6^, ^7^ The Stroke 112 video is freely available on YouTube. It won the Best Health Animation award in 2022 at the New York City International Film Festival (Figure 1).

**FIGURE 1:**
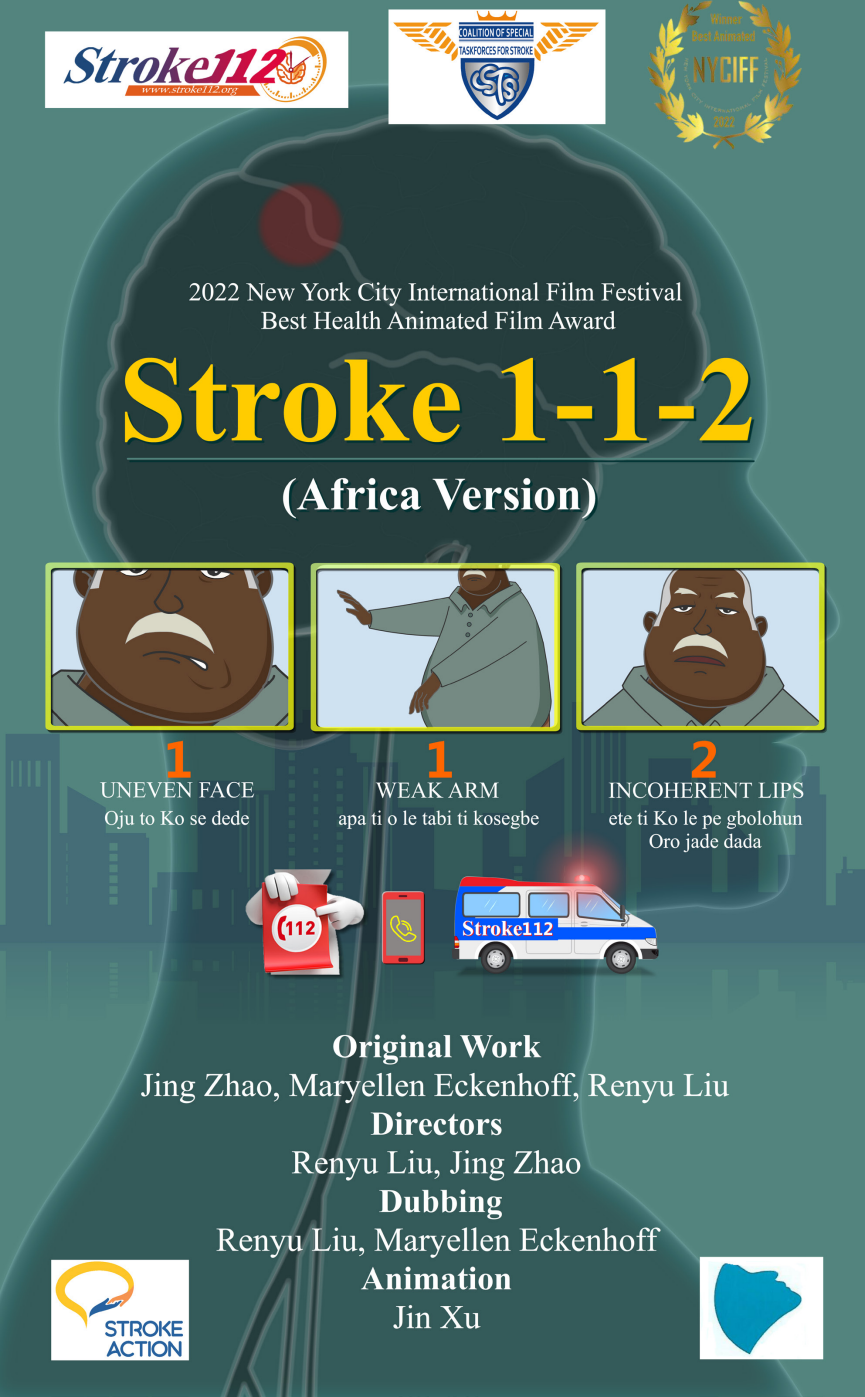
Short video poster for the award‐winning Stroke 112 animation, for Africa Countries where 112 is used as the emergency phone number. It is estimated that more than 2000 languages are spoken in the African continent, indicating significant language barriers. Using numbers to represent stroke signs and symptoms is the easiest way to promote stroke awareness across many languages, instead of FAST, the commonly used acronym for stroke education purposes in English‐speaking countries. There is no need to remember these English letters and words. Stroke 112 and the video can be translated into many local languages without losing its key messages. The Stroke 112 video is freely available on YouTube. This version has Yoruba subtitles as an example

The reality is that in much of Africa the standard of acute stroke care in institutions falls way below recommendations, and prehospital stroke care is non‐existent in most vicinities. Where emergency services are available, they are rudimentary and not likely to have the sort of impact expected for stroke care. A 2009 study in Nigeria, investigating emergency medical services available in the country, affirmed this sorry state of affairs. Their study revealed that prehospital emergency medical services were provided mainly by relatives (52.83%), security agencies (40.42%), and bystanders (6.74%).^8^

A major cause for long prehospital times is the delay in seeking medical help by the patient or by the people witnessing the stroke. Several studies in developed countries have evaluated the factors causing prehospital delays and have reported considerable delays in patients with acute stroke symptoms presenting at emergency departments.^5^, ^6^ Poor recognition of stroke symptoms and low perception of threat leads to delays in seeking medical attention after the onset of stroke symptoms, often leading to missed treatment opportunities. There are several barriers worthy of mention as leading causes to delays of treatment in Africa and much of the world. The identification of stroke symptoms amenable to treatment is paramount but has proven difficult prior to hospital evaluation. Barriers faced by emergency medical services (EMS) including patient access and communication remain challenges to overcome. Access to the appropriate level of care, especially to hospitals capable of invasive therapies, remain elusive. These challenges in Africa and other developing countries must always be kept in perspective so that innovations and solutions can continually evolve to meet and address them. Only then can the stroke dilemma (the stroke curse) be reversed.

The genesis of the Africa Stroke Organization in 2020 has provided a much needed and supportive platform to galvanize concerted actions toward changing this narrative across the continent by providing leadership, driving policy, and the implementation of stroke management protocols in and out of hospitals.^9^ The organization is committed to influencing policymakers to engage in active stroke advocacy and provide committed government investment to improve stroke care in African countries. Recent establishment of the World Stroke Organization Taskforce of Prehospital Care (WSOTPC) and the Coalition of the Special Taskforces for Stroke (CSTS) aimed to improve stroke prehospital care with a special focus on middle‐ and low‐income countries and shed some light into improving stroke care across the world.^10^

## FUNDING INFORMATION

The National Natural Science Foundation of China; CIHR, Grant/Award Number: 81973157, PI: JZ. Funding from the University of Pennsylvania; Grant/Award Number: CREF‐030, PI: RL.

## CONFLICT OF INTEREST

All authors have no conflict of interest to declare.

## Data Availability

Data are available for reseanable request to Dr. Renyu Liu via email: renyu.liu@pennmedicine.upenn.edu.

## References

[1] O'Donnell MJ , Xavier D , Liu L , et al. Risk factors for ischaemic and intracerebral haemorrhagic stroke in 22 countries (the interstroke study): a case‐control study. Lancet. 2010;376:112‐123. 20561675 10.1016/S0140-6736(10)60834-3

[2] Owolabi M , Olowoyo P , Popoola F , et al. The epidemiology of stroke in africa: a systematic review of existing methods and new approaches. J Clin Hypertens (Greenwich). 2018;20:47‐55. 29228472 10.1111/jch.13152 PMC5777902

[3] Ekenze OS , Onwuekwe IO , Ezeala Adikaibe BA . Profile of neurological admissions at the university of Nigeria teaching hospital enugu. Niger J Med. 2010;19:419‐422. 21526631 10.4314/njm.v19i4.61967

[4] Ekeh B , Ogunniyi A , Isamade E , Ekrikpo U . Stroke mortality and its predictors in a nigerian teaching hospital. Afr Health Sci. 2015;15:74‐81. 25834533 10.4314/ahs.v15i1.10 PMC4370132

[5] Zhao J , Eckenhoff MF , Sun WZ , Liu R . Stroke 112: a universal stroke awareness program to reduce language and response barriers. Stroke. 2018;49:1766‐1769. 29925649 10.1161/STROKEAHA.118.021729 PMC6034704

[6] Wang Y , Liu Y , Li D , Zheng K , Chi F , Zhao J . Recent developments in ischemic stroke prehospital care from stroke 120 special task forces. Transl Perioper Pain Med. 2021;8:342‐345.

[7] Yuan J , Li M , Liu Y , et al. Analysis of time to the hospital and ambulance use following a stroke community education intervention in China. JAMA Netw Open. 2022;5:e2212674. 35579896 10.1001/jamanetworkopen.2022.12674 PMC9115614

[8] Solagberu BA , Ofoegbu CK , Abdur‐Rahman LO , Adekanye AO , Udoffa US , Taiwo J . Pre‐hospital care in Nigeria: a country without emergency medical services. Niger J Clin Pract. 2009;12:29‐33. 19562917

[9] Akinyemi R , Sarfo F , Abd‐Allah F , et al. Conceptual framework for establishing the african stroke organization. Inter J Stroke. 2021;16:93‐99. 10.1177/1747493019897871 PMC8006214 32026763

[10] Liu R , Zhao J , Li X , Messe S , Fisher M , Rudd A . To use stroke 911 to improve stroke awareness for countries where 911 is used as an emergency phone number. CNS Neurosci Ther. 2022;28:1473‐1475. 35924380 10.1111/cns.13931 PMC9437232

